# Leaf metabolic signatures induced by real and simulated herbivory in black mustard (*Brassica nigra*)

**DOI:** 10.1007/s11306-019-1592-4

**Published:** 2019-09-28

**Authors:** Stefano Papazian, Tristan Girdwood, Bernard A. Wessels, Erik H. Poelman, Marcel Dicke, Thomas Moritz, Benedicte R. Albrectsen

**Affiliations:** 1 Department of Plant Physiology, Umeå University (Umeå Plant Science Centre), 90187 Umeå, Sweden; 2 Laboratory of Entomology, Wageningen University, 6700 AA Wageningen, The Netherlands; 3 Department of Forest Genetic and Plant Physiology, Swedish University of Agricultural Sciences (Umeå Plant Science Centre), 90187 Umeå, Sweden

**Keywords:** Metabolomics, Methyl jasmonate, *Brassica nigra*, Growth-defence allocation, Priming, Herbivore-induced responses, Leaf ontogeny, Glucosinolates

## Abstract

**Introduction:**

The oxylipin methyl jasmonate (MeJA) is a plant hormone active in response signalling and defence against herbivores. Although MeJA is applied experimentally to mimic herbivory and induce plant defences, its downstream effects on the plant metabolome are largely uncharacterized, especially in the context of primary growth and tissue-specificity of the response.

**Objectives:**

We investigated the effects of MeJA-simulated and real caterpillar herbivory on the foliar metabolome of the wild plant *Brassica nigra* and monitored the herbivore-induced responses in relation to leaf ontogeny.

**Methods:**

As single or multiple herbivory treatments, MeJA- and mock-sprayed plants were consecutively exposed to caterpillars or left untreated. Gas chromatography (GC) and liquid chromatography (LC) time-of-flight mass-spectrometry (TOF-MS) were combined to analyse foliar compounds, including central primary and specialized defensive plant metabolites.

**Results:**

Plant responses were stronger in young leaves, which simultaneously induced higher chlorophyll levels. Both MeJA and caterpillar herbivory induced similar, but not identical, accumulation of tricarboxylic acids (TCAs), glucosinolates (GSLs) and phenylpropanoids (PPs), but only caterpillar feeding led to depletion of amino acids. MeJA followed by caterpillars caused higher induction of defence compounds, including a three-fold increase in the major defence compound allyl-GSL (sinigrin). When feeding on MeJA-treated plants, caterpillars gained less weight indicative of the reduced host-plant quality and enhanced resistance.

**Conclusions:**

The metabolomics approach showed that plant responses induced by herbivory extend beyond the regulation of defence metabolism and are tightly modulated throughout leaf development. This leads to a new understanding of the plant metabolic potential that can be exploited for future plant protection strategies.

**Electronic supplementary material:**

The online version of this article (10.1007/s11306-019-1592-4) contains supplementary material, which is available to authorized users.

## Introduction

Plants are constitutively protected against herbivores by several mechanisms including physical barriers (e.g. trichomes and thorns) and chemical defences (D’Auria and Gershenzon 2005; Milkowski and Strack 2010; Onkokesung et al. 2014). Upon damage, defences are further induced by rapid metabolic reconfigurations (Karban 2011; Meldau et al. 2012). The plant metabolome is highly interconnected through enzymatic reactions of which some are responsible for the synthesis of specialized defence metabolites, such as phenylpropanoids (PPs) and glucosinolates (GSLs, Halkier and Gershenzon 2006; Morrisey 2009). Specialized (or ‘secondary’) biosynthetic metabolic pathways are taxon-specific and evolved from the central (or ‘primary’) metabolism (Firn and Jones 2009; Weng 2014; Moghe and Last 2015), which is related to growth, photosynthesis, and nitrogen assimilation, but which is also responsible for the production of precursors of specialised compounds. Most defence metabolites further depend on conjugated sugar moieties for stability, translocation, and storage inside the plant’s cellular compartments (Rask et al. 2000; Gachon et al. 2005; Le Roy et al. 2016). It is consequently well accepted that herbivore-induced responses of plants not only result in de-novo synthesis of defence compounds, but also depend on the central metabolism (Schwachtje and Baldwin 2008; Zhou et al. 2015).

Plant hormones are signalling chemicals that are present in small concentrations, e.g., auxin, cytokinins, ethylene (ET), salicylic acid (SA), and jasmonic acid (JA). They control plant growth metabolism and coordinate and integrate the metabolic crosstalk processes that direct resources between developmental and protective needs (Erb et al. 2012; Huot et al. 2014). JA and its derivatives belong to a class of oxylipins, synthesized from the octadecanoid pathway, that are particularly induced by chewing herbivores (Farmer and Ryan 1992; Huang et al. 2017; Wasternack and Song 2017). Upon herbivore attack, JA accumulates intracellularly and initiates signal transductions across tissues and organs (Lortzing and Steppuhn 2016). JA signals can travel along the phloem leading to systemic responses (Glauser et al. 2008; Mousavi et al. 2013), while the ester derivative methyl-JA (MeJA), as a volatile cue, can transfer resistance responses through air to remote plant parts and even between neighbouring plants (Farmer and Ryan 1990; Wasternack and Song 2017). Exposure to either JA or MeJA typically elicits the production of plant defence metabolites, e.g. GSLs (van Dam and Oomen 2008; Fritz et al. 2010; Zang et al. 2015; Yi et al. 2016). For these reasons, JA and MeJA are frequently applied to plants to simulate herbivory experimentally and to enhance, or prime, plant resistance (Sampedro et al. 2011; Balmer et al. 2015; Lortzing and Steppuhn 2016).

Plants continuously adjust their metabolism to account for future growth and ecological fitness opportunities (McKey 1974; Heil and Baldwin 2002; McCall and Fordyce 2010; Meldau et al. 2012). Accordingly, in response to damage, chemical defences are hypothesized to be induced and distributed following developmental needs, with higher investments in protecting young tissues compared to older ones (Brown et al. 2003; Traw and Feeny 2008; Havko et al. 2016; Ochoa-López et al. 2015; de Vries et al. 2017; Chrétien et al. 2018). Because leaf metabolism changes throughout its development—e.g. with young leaves starting as sinks to later become sources of photosynthate during maturation, leaf ontogeny may influence the plant herbivore-induced metabolic responses (Pantin et al. 2012; Townsley and Sinha 2012; Quintero et al. 2014; Ochoa-López et al. 2015; Barton and Boege 2017; Brütting et al. 2017). Thus, a combination of a plant’s external and internal signals may eventually lead to a metabolic reorganization cascade that strengthens resistance against herbivores, but which can also affect plant growth and central energy metabolism (Schwachtje and Baldwin 2008; Zhou et al. 2015; Papazian et al. 2016, Huang et al. 2017; Machado et al. 2017; Guo et al. 2018).

Metabolomics can provide detailed information about the complexity and dynamics of plant metabolism and it may detect minute responses to abiotic or biotic stress factors (Jansen et al. 2009; Khaling et al. 2015; Maag et al. 2015; Papazian et al. 2016; Peng et al. 2016; Ponzio et al. 2017). Here, we applied metabolomics to carry out an in-depth analysis of the wild black mustard plant (*Brassica nigra*) foliar metabolome in response to herbivory. Our focus was to evaluate the effects of MeJA-simulated herbivory and caterpillar feeding by the specialist herbivore *Pieris brassicae* on the metabolic response of *B. nigra*. The experimental design included plants exposed to four kinds of treatment: untreated controls (C), herbivory simulated with MeJA (M), real herbivory by caterpillars (P), and pre-treatment with MeJA followed by caterpillar feeding (MP). The effects of the treatments were evaluated in leaves of different ages to include both mature and young leaves. Specifically, the research questions set for this study were: (1) How does the MeJA pre-treatment affect the metabolism of *B. nigra* (C vs. M) and its resistance to herbivore feeding by *P. brassicae* caterpillars (P vs. MP)? (2) How does MeJA effectively mimic real caterpillar herbivory signatures in terms of the plant’s metabolic response (M vs. P)? (3) Do MeJA and caterpillar herbivory result in similar metabolomic responses in leaves of different ages (i.e. leaf ontogeny)?

We combined the application of time-of-flight mass spectrometry (TOF-MS) with gas chromatography (GC) to investigate central primary metabolism (i.e. sugars, amino acids, tricarboxylic acids, fatty acids, amines, etc.) and liquid chromatography (LC) for specialized defence metabolism (i.e. glucosinolates and phenolic compounds). Altogether, the results reported herein provide an extensive and detailed explorative map of constitutive and induced plant foliar metabolic responses to herbivory.

## Materials and methods

### Plants and insects

The herb *Brassica nigra* (Brassicaceae) and its specialist herbivore *Pieris brassicae* (Lepidoptera: Pieridae) constitute a model system of chemical ecological interactions (Blatt et al. 2008; Bruinsma et al. 2008; Broekgaarden et al. 2011; Amiri-Jami et al. 2016; Lucas-Barbosa et al. 2016). This system has been used to investigate plant adaptation to multiple stresses (Khaling et al. 2015; Kask et al. 2016; Papazian et al. 2016; Ponzio et al. 2017*).* We collected seeds of a *B. nigra* ecotype from The Netherlands (51°96′N; 5°68′) to grow in Umeå, Sweden. Seeds were vernalized (on 0.5% agarose medium, and kept in the dark for 48 h at 4 °C) to obtain uniform germination. Seedlings were planted in plastic pots (~ 73 cl) with a mixture of soil and vermiculite (3:1) and grown in the greenhouse (16:8 h light: dark cycle, 20–22 °C, 50–70% RH). Caterpillars were obtained as eggs from Wageningen University, The Netherlands (see Ponzio et al. 2017).

### Treatments and sampling

**Fig. 1:**
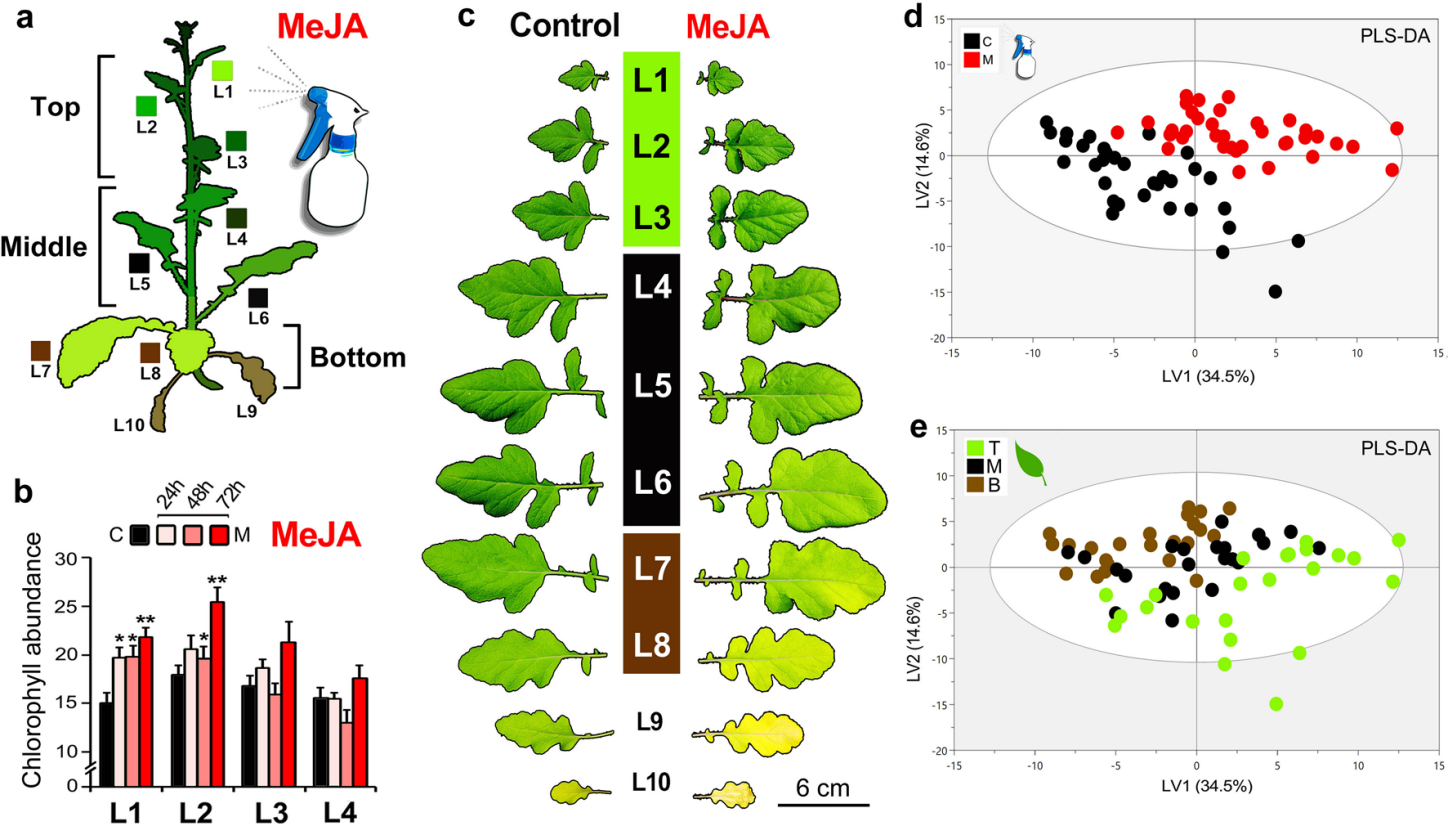
MeJA may mimic herbivory and enhance plant resistance to herbivores. MeJA (1 mM) was sprayed to whole plants of *Brassica nigra* for comparison of growth and defence related metabolic responses in MeJA-treated plants (M; n = 11) and controls (C, n = 11). **a** Foliar material was evaluated after stem position: top (T) (L1–L3), middle (M) (L4–L6), and bottom (B) (L7–L8). **b** Chlorophyll content was measured three times after treatment (with use of optical absorbance; wavelength 653 nm, mean ± SE). **c** Phenotypic leaf morphologies were characterized. **d–e** Multivariate analyses of the changes in the plant metabolome (GC–MS) detailed with the use of a PLS-DA model (two components; R^2^X cum = 49%, R^2^Y cum = 75%, Q^2^ cum = 69%). The colour codes in **d** correspond to treatment (C, M), and in **e** to leaf position (T, M, B)

**Fig. 2:**
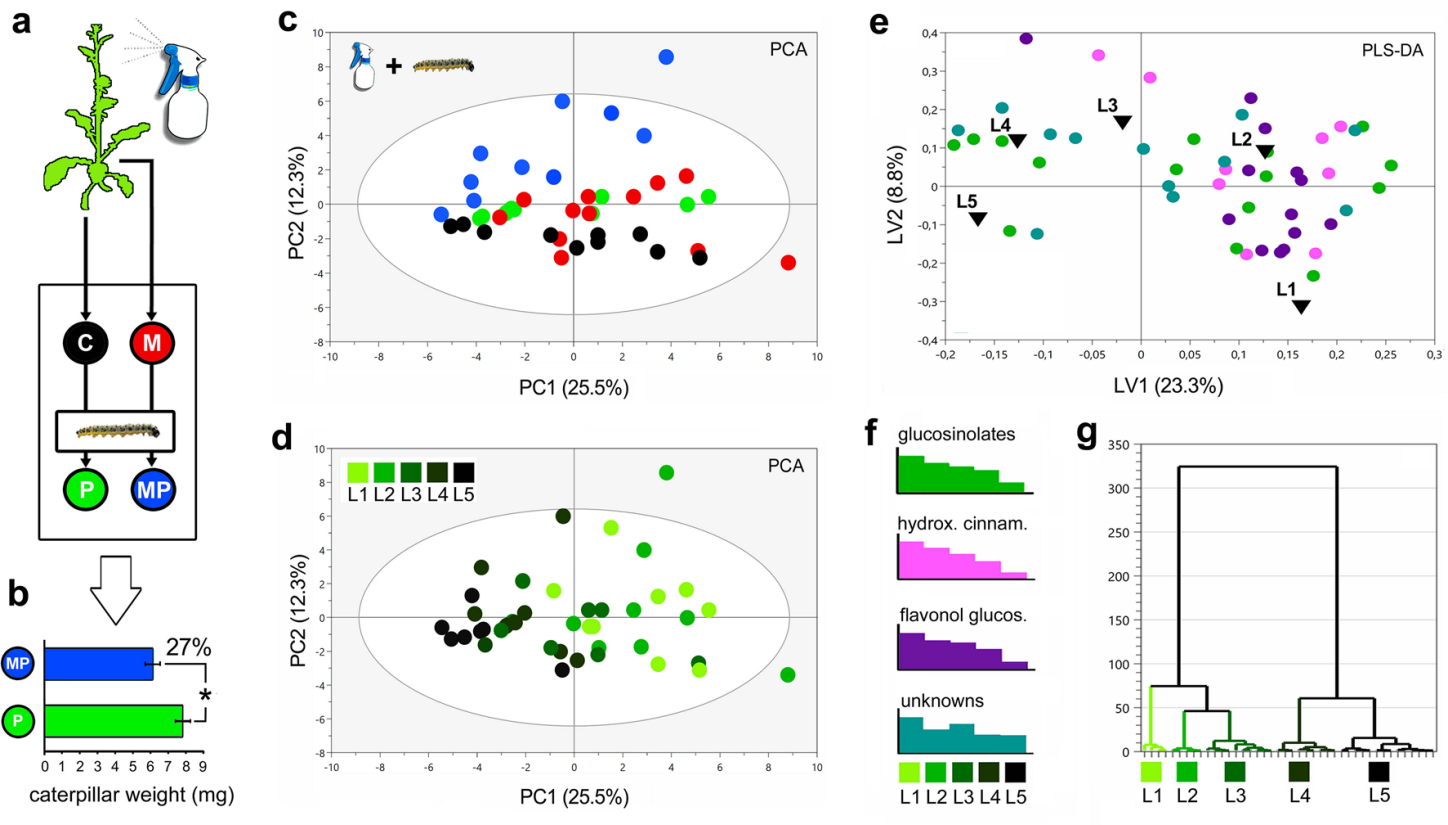
Herbivory to *B. nigra* by caterpillars of the butterfly *P. brassica* was compared to MeJA-simulated herbivory in single and combined treatments. **a** The experimental set-up included untreated controls (C) and plants sprayed with 1 mM MeJA (M). 72 h after the MeJA treatment, 15 first instar caterpillars were added to a subset of the C- and M-plants, as (P) and (MP) real herbivory treatments. **b** After 5 days of feeding, differences in caterpillar weight was assessed (Student’s *t* test, *P* value < 0.05 (*), mean (mg) ± SE). Foliar responses were characterized (LC–MS) and analyzed with **c–d** principal component analyses (PCA; five components R^2^X cum = 62%, Q^2^ cum = 16%). PCA score plot colored after **c** as an overview of responses by treatments: C (n = 10), M (n = 10), P (n = 18), MP (n = 18), and after **d** leaf positions collected for metabolic analyses (L1–L5). **e** A supervised multivariate model (PLS-DA, five components, R^2^X cum = 32%, R^2^Ycum = 30%, Q^2^ cum = 14%) detected metabolic signatures associated with leaf position. **f** The relative abundances of specialized defence metabolites were organized with the use of **g** hierarchical clustering after leaf position

Two types of treatment were used separately and in combination (Figs. 1a, 2a): simulated herbivory (M) with use of the plant hormone MeJA; real herbivory (P) by *P. brassicae* caterpillars; and combined herbivory (MP) with caterpillars feeding on M-plants for 5 days, starting at 3 days after pre-treatment with MeJA. MeJA (Sigma-Aldrich, CAS Number 39924-52-2) was applied to the whole plant by spraying 5 mL (1 mM, diluted in MilliQ water) (Gols et al. 2003). First instar caterpillars (15 individuals per plant) were placed on the first fully expanded leaf (L4–L5) of experimental plants and left to feed freely. After every experiment, leaf samples (size 5–15 cm) were collected by cutting the petiole with scissors, wrapping the harvested leaves in aluminium foil, and immediately flash-freeze them in liquid nitrogen. Samples were stored at − 80 °C until prepared for metabolomics analysis.

### Experimental set-ups

#### Experiment-1

A set of 22 healthy uniformly-sized 4-week-old plants was randomly divided into two equal-sized groups: control plants (C, 11 biological replicates) sprayed with MilliQ water as mock solution, and MeJA-simulated herbivory plants (M, 11 biological replicates). After 72 h, leaves were sampled and divided into three groups according to leaf ontogeny: young leaves from top of the plant (L1–L3), mature leaves from the middle (L4–L5), and old leaves from the bottom (L7–L8) (L#s refer to position, Fig. 1a, c). The leaf samples were prepared for analysis by GC–TOF-MS (Fig. 1d, e). Another set of plants (including both MeJA pre-treated and control plants, ten of each) were left for chlorophyll measurements (0, 24, 48, and 72 h after the MeJA application) by optical absorbance at 653 nm (CCM-200plus; Opti-Sciences^®^) and determined as the average of five technical measurements for each leaf (Fig. 1b, c).

#### Experiment-2

**Fig. 3:**
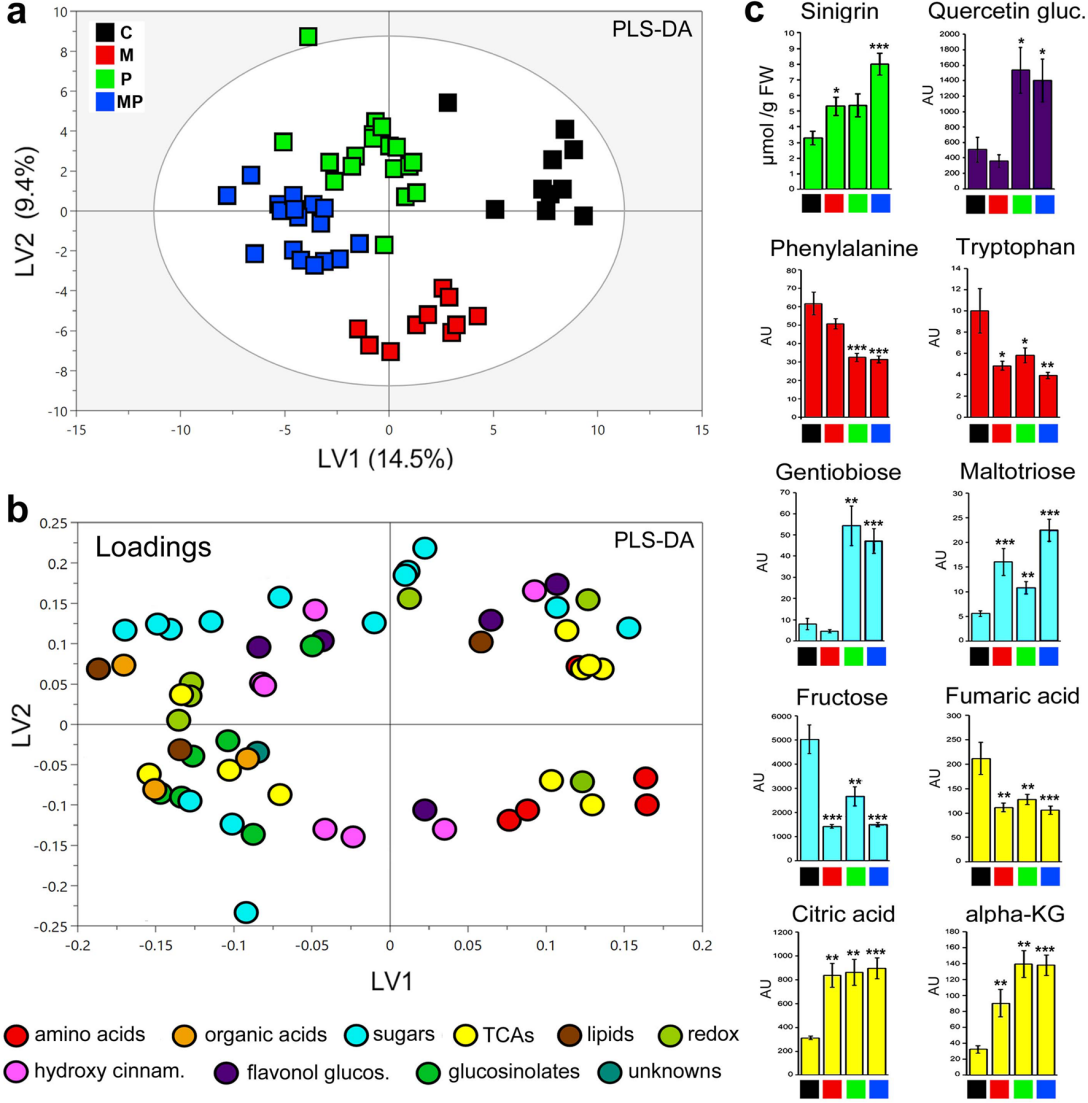
Full-metabolic responses of 4-week-old *B. nigra* plants to herbivory (mature leaves, L4-L5). Multivariate analysis for metabolomics data (GC–MS and LC–MS) are presented according to four treatments (see Fig. 2): **a** PLS-DA score plot, four component model (R^2^X cum = 42%, R^2^Ycum = 75%, Q^2^ cum = 56%) detected differences among treatments, with **b** loadings after metabolite contribution (VIP > 1.00); **c** changes in abundance of single metabolites shown as mean ± SE; Student’s *t* test, *P* values < 0.05 (*), < 0.01 (**), and < 0.001 (***). *Quercetin glucoside* = quercetin-3-sinapoylsophoroside-7-glucoside

A set of 56 uniform plants were selected from a larger cohort and randomly divided into treatment groups (Fig. 2a) between control plants sprayed with MilliQ water as mock solution, and plants sprayed with MeJA, i.e.: C- and M-plants (28 biological replicates each). After 72 h, a total of 36 plants were randomly selected out of each C- and M-group and subjected to caterpillar feeding (P- and MP, 18 biological replicates each), whereas the remaining 20 plants were kept as controls (C- and M-, 10 biological replicates each). After 5 days of feeding, the average weight of caterpillars was assessed per plant (Fig. 2b), and leaves (L4–L5) were sampled for metabolic analyses with GC– and LC–TOF-MS (Fig. 3a–c). In addition, in order to assess age-related metabolite signatures according to leaf ontogeny, individual leaves (L1–L5) were collected from another group of plants exposed to the same treatments (C, M, P, MP; two biological replicates each) and analysed with LC–TOF-MS (Fig. 2c–g).

### Metabolomics

Metabolomics analyses (GC– and LC–TOF-MS) and data processing were performed as described in Papazian et al. (2016). Leaf samples were ground in liquid nitrogen and stored at − 80 °C until extraction. Each sample (10–12 mg) was extracted in 1 ml of cold chloroform:methanol:H_2_O (20:60:20 v/v), including 7.5 ng/µl of the isotope-labelled internal standards (IS) salicylic acid-D4 (SA-D4), succinic acid-D4, glutamic acid-^13^C5,^15^N, and glucose-^13^C6. Extracts were agitated with a 3 mm tungsten carbide bead for 3 min and centrifuged at 20,800×*g* for 10 min at 4 °C.

From 1 ml initial extraction volume, approximately 800 μl of the supernatant were recollected, of which 600 μl were kept as stock samples (stored at − 80 °C) and 200 μl were evaporated to dryness using a SpeedVac and prepared for GC– and LC–TOF-MS analyses. Additionally, quality control (QC) samples were prepared from the stock samples with equal aliquots of 50 μl from each of the treatments (C, M, P, MP; total 200 μl) and used for method optimization and spectral quality assurance.

#### GC–TOF-MS

Dried extracts were derivatized with methoxyamine and *N*-Methyl-*N*-(trimethylsilyl) trifluoroacetamide (MSTFA) (Gullberg et al. 2004). Samples were analysed by GC–TOF-MS (on an Agilent 6890 gas chromatograph equipped with a 10 m × 0.18 mm silica capillary column with a 0.18 µm DB 5-MS UI stationary phase, J&W Scientific; connected to a LECO Pegasus III TOF-MS) operated using the LECO ChromaTOF^®^ software package (Leco Corp., St Joseph, MI, USA). Split-less injections of 2 µL (two technical replicates for each sample) were performed by a CTC Combi Pal autosampler (CTC Analytics AG, Switzerland). The injector temperature was set to 270 °C, the purge flow rate to 20 ml min^−1^. The gas flow rate through the column was 1 ml min^−1^, the column temperature was held at 70 °C for 2 min, then ramped by 40 °C min^−1^ to 320 °C, and finally held for 2 min. The transfer line and the ion source temperatures were 250 °C and 200 °C, respectively. Ions were generated by a 70 eV electron impact beam at an ionization current of 2.0 mA, and 30 spectra s^−1^ were recorded in the mass range 50–800 *m/z*. The acceleration voltage was turned on after a solvent delay of 150 s. The detector voltage was 1500–2000 V. Technical variation was measured as the relative standard deviation (RSD%) in spectral intensities of the isotope-labelled IS (succinic acid-D4, glutamic acid-^13^C5,^15^N, and glucose-^13^C6, and SA-D4) and of a post-extraction procedural standard (methyl stearic acid), and was used to normalize the metabolite peak intensities.

#### LC–TOF-MS

Dried extracts were re-dissolved in 20 µl methanol:water (50:50 v/v) and analysed with an UHPLC-ESI-TOF-MS (Waters, Milford, MA USA) operated with MassLynx™ v. 4.1 software (Waters, Milford, MA, USA). The LC Acquity™ system was equipped with a 2.1 × 100 mm, 1.7 μm C18 UPLC™ column (held at 40 °C) and coupled to an LCT Premier TOF-MS. Two injections of 2 µl were analyzed for each individual sample as technical replicates. Gradient elution was performed using solvent mobile phases A (water + 0.1% formic acid) and B (acetonitrile + 0.1% formic acid), gradually increasing the proportion of solvent B over time: 0 to 4 min, 1% to 20% B; 4 to 6 min, 20% to 40% B; 6 to 9 min, 40% to 95% B; and 9 to 13.5 min, 95% B. The total running time for each sample was 19 min, with a flow rate of 500 μl min^−1^. The source temperature was 120 °C, cone gas flow was 10 l h^−1^, desolvation temperature was 320 °C, nebulization gas flow was 600 l h^−1^, and capillary and cone voltages were set at 2.5 kV (negative ionization mode) and 35 V, respectively. The system was operated in dynamic range enhancement (DRE) mode. For accurate mass measurements, the lock mass compound leucine enkephalin (Leu-enk) was infused at 400 pg μl^−1^ in 50:50 acetonitrile:water at 20 μl min^−1^. The normal lock mass used in DRE mode was the negative ^13^C ion of Leu-enk (*m/z* 555.265), and the extended lock mass was the normal negative ion (*m/z* 554.262). Mass spectra were acquired in centroid mode with an *m/z* range of 100 to 1000, and the data threshold value was set to 3. Technical variation was measured as the RSD% in spectral intensities of the isotope-labelled IS and was used in order to normalize the identified metabolite peak intensities.

### Data processing and identification

Data processing, including peak alignment, integration and feature extraction, were performed using a Matlab^®^ script developed in-house at the SMC. Instrumental variation of the MS signal during GC–MS and LC–MS data acquisition was monitored and corrected by normalizing sample intensities on the integrated areas of the internal standards. Retention indices (RIs) of compounds detected with GC–MS were calculated relative to those of a C8–C40 alkane series. Metabolite identification was achieved by matching mass-spectra and RIs to the in-house SMC library combining automated peak deconvolution and targeted analysis (versus a predefined list of RI windows and *m/z* values) and to the public Golm Metabolome Database developed by the Max Planck Institute (http://gmd.mpimp-golm.mpg.de). For comparison with the Golm Metabolome Database, RIs measured on the 5% phenyl–95% dimethylpolysiloxane capillary column VAR5 (Golm Metabolome Database) were transferred to the DB-5 (10 m) system of the UPSC-SMC (Strehmel et al. 2008; Hummel et al. 2010). For identification of compounds detected with LC–MS, spectra were compared to those of pure GSL standards of sinigrin, glucobrassicin, gluconapin, glucotropaeolin, gluconasturtiin, and sinalbin (Phytoplan, Diehm and Neuberger GmbH, Heidelberg, Germany), and literature references for GSLs (Clarke 2010) and PPs, i.e. hydroxycinnamic acid derivatives and flavonol glucosides (Lin et al. 2011). Tandem mass data analysis from UHPLC-LTQ-Orbitrap (Thermo Fisher Scientific) were used to compare the spectra profiles and further confirm the identifications (Papazian et al. 2016).

### Statistical analysis

Multivariate analysis was performed in SIMCA^®^ 14 (Umetrics, Umeå, Sweden) and Minitab 17 Statistical Software^®^ 2010 (State College, PA: Minitab, Inc.) was used for univariate analyses. These analyses were of exploratory nature with the aim of generating new hypotheses, and consequently *P* values were not adjusted for multiple responses (Rothman 1990).

## Results

Combining GC- and LC–TOF-MS metabolomics allowed a comprehensive coverage of the metabolic changes in black mustard (*B. nigra*) induced upon herbivory. A total of 412 features were detected by GC–MS, of which 103 were identified as central primary metabolites (including carbohydrates, TCAs, amino acids, fatty acids, amines, etc.), based on the matching of the respective metabolite RIs and mass-spectra to the reference libraries (see Materials and methods—Sect. 2.5). LC–MS analysis detected 260 features. By comparing spectral information from literature with analyses of pure compound standards (see Materials and methods—Sect. 2.5) a total of 42 metabolites were identified as different classes of specialized defence compounds from *Brassica* sp. These included GSLs (Clarke 2010) of which the most abundant was allyl-GSL (sinigrin), and several PPs, including hydroxycinnamic acid derivatives, sinapic acid esters, and flavonol glucosides (Lin et al. 2011). Technical variation throughout the analyses (measured as the RSD% in spectral intensities of isotope-labelled IS; see Materials and methods—Sect. 2.4) was on average lower for GC–MS (11%) than for LC–MS (17%) and within the ranges expected for MS-based metabolomics analyses—i.e. < 10–20% (Parsons et al. 2009). The biological metabolic variation (measured as the RSD% in spectral intensities across all identified metabolites) differed in magnitude depending on the metabolite taken into consideration, but it was generally lower for central primary metabolism (31–62%) compared to specialized defence secondary metabolism (77–82%). Compared to untreated control plants, the metabolic variability increased when plants were exposed to herbivory (+ 5–20%), and in young leaves compared to older leaves (+ 15–30%) (Figs. 1, 2, and Supplementary dataset).

### MeJA induction (M vs. C)

MeJA caused considerable visual (Fig. 1a–c, and Fig. S1) and metabolic (Fig. 1d, e) phenotypic changes to *B. nigra*. Multivariate analyses of the GC–MS profiles (Fig. 1d, e) suggested an increase of many central metabolites in M-plants compared to C-plants. Monosaccharides (glucose, glucose 6-P, and fructose-6-P), polysaccharides (trehalose and maltose), and C_5_-C_6_-intermediates of the tricarboxylic acid (TCA) cycle generally increased, whereas fructose, sorbose, and fumaric acid (a C_4_-TCA intermediate) decreased relative to controls (Table S1). Almost no changes were detected for amino acids, apart from minute changes related to leaf position in aspartic acid and leucine that increased, and phenylalanine that decreased after M-treatment. Young leaves displayed the strongest metabolic responses (Fig. 1d, e, Table S1). The M-treatment further induced dark pigmentation of the main stem (Fig. S1), likely associated with altered changes in PPs (e.g. phenolic precursors shikimic, caffeic, and salicylic acids, Table S1). MeJA also caused changes to leaf chlorophyll, depending on leaf position, with higher levels found particularly in young (darker) M-leaves (L1–L3, Fig. 1b), whereas older M-leaves (> L5) displayed symptoms of accelerated senescence, compared to untreated controls (Fig. 1c). In summary, morphological and metabolic responses to MeJA indicate that the induced phenotypic changes extended beyond the activation of defence priming (Hilker et al. 2016; Martinez-Medina et al. 2016).

### Simulated herbivory and caterpillar feeding (M vs. P)

Metabolic analyses in *B. nigra* showed similarities between inductions in M- and P-plants, in particular, for specialized defence compounds in young leaves (L1–L3, Fig. 2c–g). GSLs, e.g. sinigrin, gluconapin, glucotropaeolin and neoglucobrassicin (detected with LC–MS) were always upregulated, sinapic acid esters (e.g. disinapoyl-gentiobiose, 1,2-disinapoyl-glucoside, and sinapoylferuloylgentiobiose) also increased, and hydroxycinnamic acid derivatives (e.g. *p*-coumaroyl-d-glucose, 1-caffeoyl-β-glucose and 1-O-sinapoyl-glucose) decreased (Table 1). Sugar levels (detected with GC–MS) were strongly affected in both M- and P-plants, with major declines of monosaccharides and sugar alcohols in the mature leaves (L4–L5). The JA precursor α-linolenic acid dropped both in M- and P-plants compared to controls, while C_5_-C_6_ TCAs (e.g. citric acid, α-ketoglutaric acid) increased, and C_4_ intermediates (succinic, malic, and fumaric acid) decreased (Fig. 3a–c and Table 1). The redox and antioxidant metabolism were affected as evidenced by the decrease in glutathione (GSH and GSSG) in both M- and P-plants. Although M- and P-plants showed similar metabolic profiles, responses were not identical for all metabolites. For instance, in M-plants, sugars such as glucose, fructose, sorbose and *myo*-inositol decreased more strongly compared to the responses observed in P-plants. Instead, gentiobiose, ascorbic and dehydroascorbic acid increased only in response to caterpillar herbivory (in P-plants). Other differences between the metabolic signatures of M- and P-plants included changes in amino acid levels with reduction of tryptophan and GABA in M-plants and lower levels of aspartic acid, glutamine, phenylalanine, and tryptophan in P-plants than in M-plants. The flavonol quercetin-3-sinapoylsophoroside-7-glucoside was induced to higher levels only in P-plants (Fig. 3c, Table 1). In conclusion, while the metabolic responses of plants induced by simulated herbivory (M) and real caterpillar feeding (P) were similar for a majority of metabolites (including TCA cycle and GSL metabolism), the two treatments also caused individual metabolic signatures.

**Table 1:**
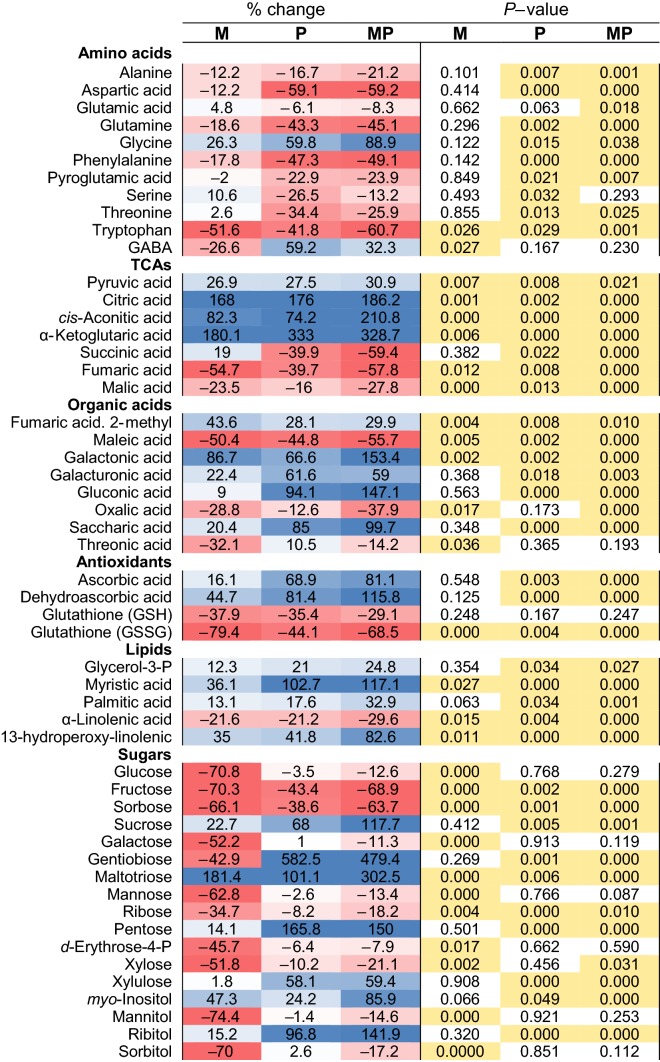

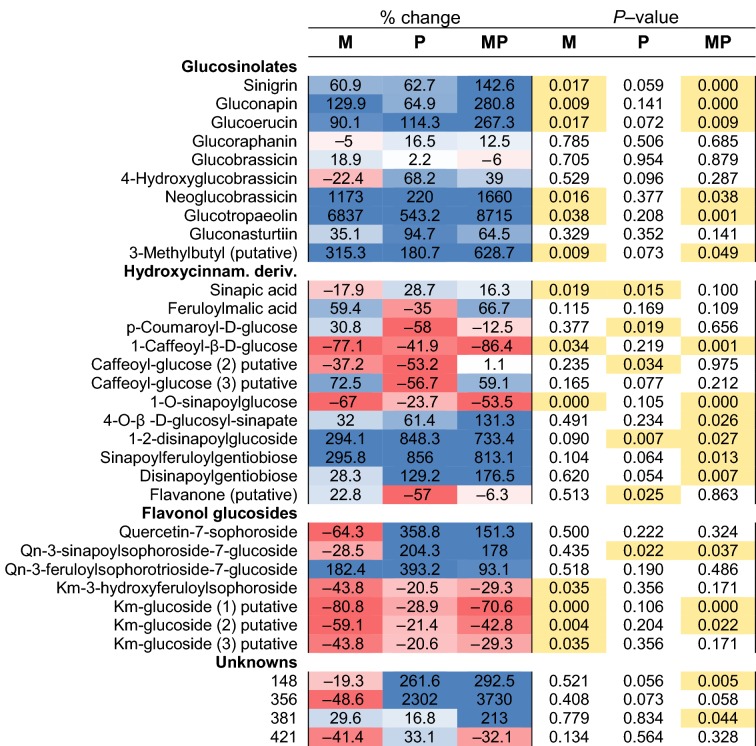
Growth and defence inductions in mature leaves

Metabolic changes (GC– and LC–TOF-MS) measured in *B. nigra* mature leaves (L4–L5) after MeJA application (M; 72 h treatment), caterpillar herbivory (P, 120 h treatment), or sequential treatment (MP, 72 + 120 h). Changes are percentage (%) of controls. Blue and red colors indicate up- and down- regulation. Significant *P* values (< 0.05, two-tailed Student’s *t* test) are highlighted in yellow. See Fig. 3

### Enhanced resistance of M-plants (P vs. MP)

Enhanced resistance against *P. brassicae* caterpillars was confirmed by caterpillar weight, which was 27% less after 5 days of feeding on M-plants (MP) compared to controls (P) (MP = 6.14 ± 1.86 mg, vs. P = 7.82 ± 1.97 mg; *t* test *P* value < 0.05, df = 34; Fig. 2a, b). Foliar metabolic defence responses were induced in both P- and MP-plants, but often more strongly in MP-plants (Figs. 2c–g, 3a–c). GSLs increased: sinigrin (two-fold from ca. 5 to 9 µmol/g FW in MP-plants; *t* test; *P* value < 0.001; df = 26), gluconapin, glucoerucin, neoglucobrassicin and glucotropaeolin (from two to 17-fold higher responses in MP-plants compared to P-plants, Table 1). PPs (hydroxycinnamic acid esters and flavonol glucosides) followed similar dynamics and increased both in P- and MP-plants, although some sinapoyl and flavonol glucosides increased more strongly in P-plants. Other sugars such as ribitol, maltotriose and gentiobiose (a conjugated moiety of many flavonol glucosides) always increased after herbivory (in P- and MP-plants). In addition, one unidentified compound (‘381’, *m/z* [M–H]^−^ 349.145) that had previously been related to biotic stress responses in *B. nigra* (Khaling et al. 2015; Papazian et al. 2016) was elevated in MP-plants (Table 1), suggesting that it is involved in protective metabolism in this study system.

## Discussion

**Fig. 4:**
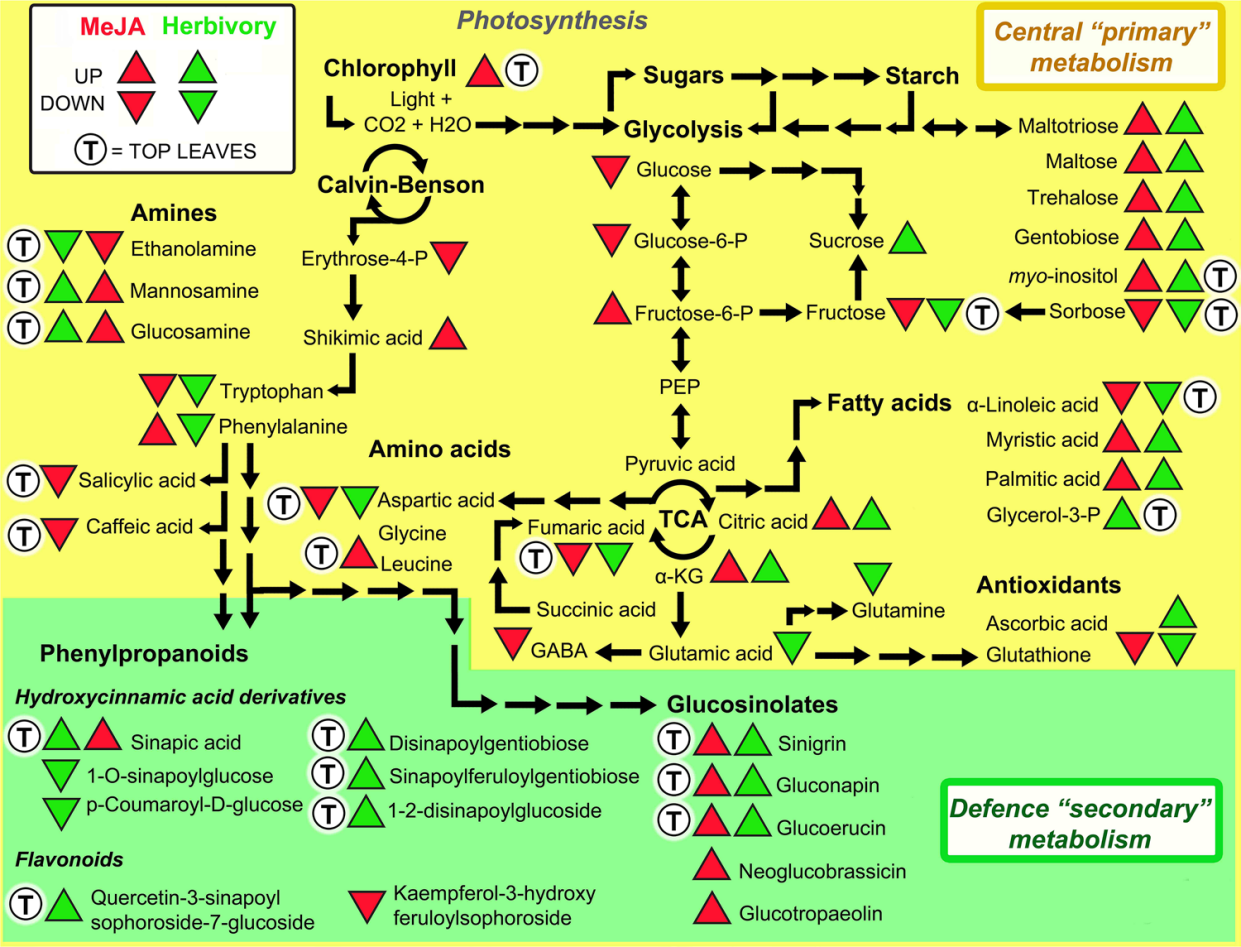
Metabolic signatures of herbivore induction mapped for *Brassica nigra*. Model suggesting mechanisms behind induced metabolic allocation effects in leaves of *B. nigra* after exposure to MeJA-simulated herbivory (red triangles) and caterpillar damage (green triangles). The direction of the triangles symbolizes metabolic enhancement (up) and reduction (down). Metabolites are divided into growth related compounds (yellow background) belonging to the central (or “primary”) metabolism (e.g. glycolysis, TCA cycle, amino acids, sugars, fatty acid metabolism, etc.) and into defence related metaboliltes (green background) belonging to the specialized (or “secondary”) metabolism. The strongest responses to the treatments of this study were found for young top leaves (T) that simultaneously increased chlorophyll levels (Figs. 1, 2). In summary, MeJA-simulated herbivory and caterpillar feeding both induced energy-fuelling reconfiguration of metabolic precursors along central pathways (such as TCA cycle intermediates) to sustain induction of defences (PPs, GSLs), with the strongest responses found in young top leaves

The plant hormone MeJA is used experimentally to simulate herbivory and enhance plant defence metabolism. We employed metabolomics to compare foliar constitutive chemistry and induced responses of *B. nigra* after treatment with MeJA and caterpillar (*P. brassicae*) herbivory. In addition to morphological changes, MeJA induced a change in the metabolic phenotype. MeJA strongly elicited plant defence metabolism when compared with control plants. Deviations in the metabolome between responses to MeJA and herbivory suggested that MeJA does not perfectly simulate herbivory by *P. brassicae*, yet MeJA pre-treatments enhanced plant resistance by reducing caterpillar weight. In all cases, our results point at strong effects of leaf ontogeny suggesting that younger leaves are metabolically more responsive than older leaves and thus potentially better protected (Fig. 4).

### MeJA induces the entire metabolome

MeJA caused visual changes in *B. nigra* that were in agreement with foliar phenotypes previously observed in other systems (Ananieva et al. 2007; Ding et al. 2018; Li et al. 2018). The resulting changes in the foliar metabolome, altered by MeJA were not only relevant for plant defences but also for primary growth, for instance the TCA metabolism. As expected from previous studies (Dombrecht et al. 2007;van Dam and Oomen 2008; Zang et al. 2015; Yi et al. 2016), levels of GSLs and PPs also increased. Other studies have also shown suppression of herbivore growth in response to either JA-treatment of their food plant or the plant previous exposure to herbivory (Albrectsen et al. 2004; Zhang and Turner 2008; Campos et al. 2016; Machado et al. 2017), but rarely have these effects been treated simultaneously and with detailed metabolomics insight as in this present study (Fig. 4).

The TCA cycle provides a central link between plant growth and defence (Fig. 4), because it generates precursors for both primary and secondary metabolic pathways (Tschoep et al. 2009; Sweetlove et al. 2010; Foyer et al. 2011). The metabolic flux through the TCA pathway is sensitive to the plant physiological status (Bolton 2009; Araujo et al. 2012) and can switch between a complete (‘*closed*’) and an incomplete (‘*open*’) cycle depending on photosynthetic opportunities (Gardeström et al. 2002; Sweetlove et al. 2010; Igamberdiev and Eprintsev 2016). Consequently, when *B. nigra* was stressed with MeJA in this study, regulation of the TCA cycle and the sugar metabolism pinpoints the high energy demand required to fuel the *de*-*novo* biosynthesis of defence compounds (Bolton 2009).

Overall, our data support that MeJA-induced resistance is a result of multiple factors with combined effects at several levels of metabolic regulation. These effects are likely a reflection of plant growth-defence hormone cross-talks. Cross-talk between JA and the growth related hormone ethylene (ET) are well known responses to herbivory. We confirmed such synergies by conducting a study of MeJA-application in *Arabidopsis*-mutants (Fig. S2), showing that while MeJA induces the expression of the defence master regulator *MYC2* transcription factor (Lortzing and Steppuhn 2016), it also interacts with ET signalling via the leaf senescence regulator *EIN3* (*ETHYLENE INSENSITIVE 3*) (Li et al. 2013; Song et al. 2014).

### MeJA partly simulates caterpillar herbivory

Simulated (MeJA) and caterpillar (*P. brassicae*) herbivory induced similar changes to the metabolome important for defence, growth, and development (Schwachtje and Baldwin 2008; Zhou et al. 2015). In the model *Arabidopsis,* GSL biosynthesis is estimated to cost more than 15% of the energy provided by photosynthesis (Bekaert et al. 2012). In this context, one of the most important insights from our study was that, in addition to a similar induction of GSLs, MeJA-simulated herbivory and real caterpillar feeding both induced the same energy-fuelling reconfiguration of TCA cycle intermediates, with an increase in citric and α-ketoglutaric acids, and a decrease in fumaric acid (Bolton 2009; Igamberdiev and Eprintsev 2016; Pastor et al. 2014; Balmer et al. 2018).

Both physical and chemical factors shape how plants and herbivores interact with each other. Enzymes and elicitors contained in the oral secretions of caterpillars and in disrupted plant tissues (i.e. damage associated molecular patterns, or DAMPs) can interfere with the plant metabolism and defence signaling, leading to suppression or amplification of the defence responses (Mattiacci et al. 1995; Reymond et al. 2000; Consales et al. 2012, Klauser et al. 2015). In cotton, Eisenring et al. (2018) for example found expected effects of herbivore feeding mode when chewing herbivores induced mainly the JA- and abscisic acid pathway, whereas sucking aphids decreased the levels of SA and suppressed the JA pathway. Interestingly, in our study, we found several PPs and flavonols increasing only in response to real herbivory (e.g. sinapic acid, disinapoyl-gentiobiose, and quercetin-3-sinapoylsophoroside-7-glucoside), whereas at the level of central metabolism MeJA and herbivory had different effects on sugars (e.g. sucrose, maltotriose, gentiobiose, *myo*-inositol). Moreover, only herbivory by caterpillars negatively affected the level of almost all amino acids, while MeJA only affected tryptophan (indolic GSLs precursor; Halkier and Gershenzon 2006) and had no effect on phenylalanine (aromatic GSLs and PPs precursor).

Overall, MeJA could mimic many of the effects of real caterpillar herbivory for a large subset of metabolites, although it did not induce an identical response. These discrepancies between the metabolic responses elicited by MeJA and caterpillars may possibly rise from the physical interaction between the herbivore and the host plant.

### MeJA enhances plant resistance to caterpillars

MeJA ability to enhance plant responsiveness to biotic stress (Lortzing and Steppuhn 2016) may offer a sustainable alternative to the use of conventional plant protection chemicals (Ahn et al. 2005; Beckers and Conrath 2007; Berglund et al. 2016; Hamada et al. 2018). In this study, MeJA indeed appeared to improve plant resistance when exogenously sprayed on plants of *B. nigra*. Caterpillars that fed on plants pre-treated with MeJA gained 27% less body mass compared to controls in a no-choice experiment for 5 days.

In studies of plant–herbivore interactions and chemical ecology, the impact on a fitness-related trait like weight is often used as a proxy to assess the fitness impact caused by specific metabolites. However, such results have often been performed on basis of the detection of only a few single defence compounds, and not like in this study for the entire metabolome. MeJA-enhanced resistance can happen via induction of plant defences or via more subtle mechanisms of defence priming, i.e. a memory of a previously experienced stimuli which modifies the plant response and prepares it against a future attack (Balmer et al. 2015; Hilker et al. 2016; Martinez-Medina et al. 2016; Mauch-Mani et al. 2017). Defence metabolites (especially GSLs) in this study showed induction in the MeJA pre-treated plants following sequential herbivore-attack. An unidentified compound ‘381’ previously considered to shape defence against herbivores in *B. nigra* (Khaling et al. 2015; Papazian et al. 2016) was also induced by herbivory only following the MeJA pre-treatment. Other single compounds, e.g. a quercetin glucoside (Fig. 3c) were silent in response to the initial MeJA pre-treatment, but except ‘381’ for no compound did we find evidence of an initial silent response to (M) combined with enhanced induction upon herbivory (MP), as required in defence priming (Hilker et al. 2016; Martinez-Medina et al. 2016).

Degree of specialism is also important for the defence response to herbivores (Ali and Agrawal 2012), and because inductions after herbivory depend on the herbivore, the induced profile will also determine future herbivore attraction (Poelman et al. 2010). GSLs deter mainly generalist herbivores (Ali and Agrawal 2012; Moore et al. 2014) whereas specialist herbivores like *P. brassicae* are attracted to high sinigrin levels which they can detoxify (Winde and Wittstock 2011). The observed increase of sinigrin and of other low abundance GSLs therefore cannot explain the inferior caterpillar performance on M-plants (Fig. 3a, b). After herbivory, MP-plants also experienced increases of PPs (e.g. sinapic acid esters) and flavonols (e.g. quercetin glucosides) that present both anti-nutritive and cell-wall fortifying properties (Glauser et al. 2008; Fritz et al. 2010; Milkowski and Strack 2010; Onkokesung et al. 2014). Anti-nutritive enzymes such as proteinase inhibitors, which we did not investigate, could have had similar effects (Farmer and Ryan 1990; Leo et al. 2001). Similarly to previous transcriptomics approaches (Reymond et al. 2004) a future metabolomics study that compares induction profiles caused by generalist and specialist herbivores might allow to specifically dissect plant evolutionary conserved JA-responses from herbivore-specific components (Fig. 4).

A focal component of the MeJA pre-treatment was the effect on central metabolism, and particularly on the TCA cycle (Fig. 4). Fumaric acid is known to reflect plant metabolic and physiological complexity by playing multiple roles, from fuelling cellular respiration to functioning as alternative carbon sink for photosynthate (Araujo et al. 2012). In our study, fumaric acid initially displayed a negative correlation with constitutive levels of defence specialized metabolites, e.g. GSLs (sinigrin, gluconapin, 4-hydroxyglucobrassicin) and PPs (*p*-coumaroyl-glucose and quercetin-3-sinapoyl-7-glucoside), but during the plant response to MeJA it was quickly depleted and its levels were positively correlated with induction of defences in M- and MP-plants (Fig. S3 and Table S4). Interestingly, TCAs such as fumaric and citric acids have been shown to be central primary metabolic targets of defence priming, and their exogenous application to mediate enhanced plant defences (Pastor et al. 2014; Balmer et al. 2018).

### Induction is strongest in young leaves

In this study, both MeJA- and herbivore-induced metabolic reconfigurations were greatly influenced by leaf development. The most active responses were measured in top young leaves, at the level of both central primary metabolism, e.g. fumaric acid, fructose, sorbose (Fig. 1d, e, Table S1) and specialized defences, e.g. GSLs and PPs (Fig. 2c–g). Plants administer limited resources not only in response to external stress (Townsley and Sinha 2012; Ochoa-López et al. 2015; Havko et al. 2016) but also to balance internal allocations during leaf development (Pantin et al. 2012; Havko et al. 2016; Brütting et al. 2017; Chrobok et al. 2016; Law et al. 2018). Leaf herbivores can select leaves after position and ontogeny. In the Brassicaceae plant family, the specialist herbivore *P. brassicae* commonly lay eggs on vegetative mature leaves, whereas young caterpillars climb to the top of the plant to feed on young leaves (and later buds and flowers) that contain the highest levels of GSLs. In *B. nigra,* 90% of the GSL content consists of sinigrin (Lankau and Strauss 2007), which accumulates in high-value reproductive organs and young leaves (Smallegange et al. 2007; Lucas-Barbosa et al. 2013). Allocation of defences to the most valuable tissues—for example to young leaves high in the canopy capturing most of the light—are illustrative of the optimal defence theory (Fagerstrom et al. 1987). Herbivore preference for young leaves has been shown to negatively affect fitness of *B. nigra* plants (de Vries et al. 2018), especially when plants under attack by herbivores simultaneously compete for light with neighbouring plants (de Vries et al. 2018, 2019). Overall, our study supports that JA-induced responses result in an extensive reconfiguration of the entire plant metabolome that is largely shaped by leaf development. Consequently, both spatial and temporal specific considerations about sampling of foliar material (or multiple tissues, such as flowers and buds; see Barton and Boege 2017) are indeed necessary in this kind of experiments to accurately capture the full metabolome capacity of plant herbivore-induced defences.

## Conclusions

Metabolomics was applied here to study plant (single and multiple) exposure to MeJA-simulated and real caterpillar herbivory. We have shown that a single exogenous application of the hormone MeJA can quickly induce a reconfiguration of plant metabolism resulting in increased resistance against a specialist insect herbivore. However, we also showed how MeJA alone does not completely mimic the same metabolic signatures induced in the plant by real caterpillar herbivory. Combination of GC– and LC–MS revealed how the herbivore-induced responses can overlap between different metabolic pathways, thus highlighting the important role of both central primary and specialized secondary metabolism in plant defence to biotic stresses. Plant anti-herbivore defence responses become even more complex when we include the leaf development aspect. By measuring responses at different leaf ages, our data showed that younger leaves display the highest metabolic plasticity. This supports the hypothesis that plant metabolomic changes are induced more strongly in the most valuable tissues, as young leaves simultaneously represent a future source of photosynthate and have a high likelihood of being attacked by herbivores. Because this result was consistently observed across both primary and secondary metabolic pathways and after exposure to either MeJA-simulated or real caterpillar herbivory, it suggests that the ontogenic trajectory of plant defences is tightly coordinated throughout the plant development and partly regulated by JA-dependent mechanisms.

In conclusion, considering the plant metabolome in its entirety (rather than targeting specific classes of metabolites) and evaluating herbivore-induced responses across ontogeny (rather than single tissues) will help researchers in plant physiology and ecology to better understand the metabolic links and molecular mechanisms that make plants able to fine-tune and carefully balance growth and defence. Once applied, this knowledge can help us to fully exploit the plant metabolic potential in future plant breeding and protection strategies.

## Electronic supplementary material

Below is the link to the electronic supplementary material.

Supplementary material 1 (PDF 981 kb)

Supplementary material 2 (XLSX 415 kb)

## Data Availability

The metabolomics data reported in this paper are available as dataset in the supplementary material 2. In addition, GC–MS raw files (cdf format) were deposited to the National Institutes of Health (NIH) Common Fund’s Metabolomics Data Repository and Coordinating Center (NIH grant U01-DK097430) website, the Metabolomics Workbench (http://www.metabolomicsworkbench.org) with the project ID PR000759 (10.21228/M8K67W). LC–MS raw files (mzML format) were deposited to the NIH-funded Center for Computational Mass Spectrometry—MassIVE (https://massive.ucsd.edu) where it has been assigned the study identifier MSV000084183 (10.25345/C5TQ06).

## Abbreviations

ET: Ethylene
GSL: Glucosinolate
PP: Phenylpropanoid
GC: Gas chromatography
IS: Internal standard
JA: Jasmonic acid
LC: Liquid chromatography
MeJA: Methyl jasmonate
SA: Salicylic acid
TOF-MS: Time-of-flight mass spectrometry
TCA: Tricarboxylic acid
