# Supplementary material for: Leaf metabolic signatures induced by real and simulated herbivory in black mustard (Brassica nigra)
Source: Metabolomics. 2019 Sep 28;15(10):130. doi: 10.1007/s11306-019-1592-4 (PMC6765471; doi:10.1007/s11306-019-1592-4)
Supplement: Supplementary file 1 — Supplementary material 1 (PDF 981 kb) [file 11306_2019_1592_MOESM1_ESM.pdf]

Supplementary material for:

Metabolic signatures of herbivore induction mapped for black mustard (*Brassica nigra*)

Stefano Papazian[1], Tristan Girdwood[1], Bernard A. Wessels[1], Erik H. Poelman[2], Marcel Dicke[2], Thomas Moritz[3], Benedicte R. Albrectsen[1]

[1]Department of Plant Physiology, Umeå University (Umeå Plant Science Centre), 90187 Umeå, Sweden.

[2] Laboratory of Entomology, Wageningen University, 6700AA Wageningen, The Netherlands.

[3] Department of Forest Genetic and Plant Physiology, Swedish University of Agricultural Sciences (Umeå Plant Science Centre), 90187 Umeå, Sweden.

Email contacts:

Stefano Papazian	stefano.papazian@umu.se	ORCID: 0000-0003-2538-8702
Tristan Girdwood	tristan.girdwood@gmail.com	
Bernard Wessels	bernard.wessels@umu.se	ORCID: 0000-0003-0717-1630
Erik H. Poelman	erik.poelman@wur.nl	ORCID: 0000-0003-3285-613X
Marcel Dicke	marcel.dicke@wur.nl	ORCID: 0000-0001-8565-8896
Thomas Moritz	thomas.moritz@slu.se	ORCID: 0000-0002-4258-3190

Corresponding author:

Benedicte R. Albrectsen	benedicte.albrectsen@umu.se	ORCID 0000-0002-9337-4540
	Tel: +46 703384919	

Supplementary Figures and Tables **p. 2**

Supplementary Methods **p. 9**

References **p. 11**

Supplementary Figures and Tables

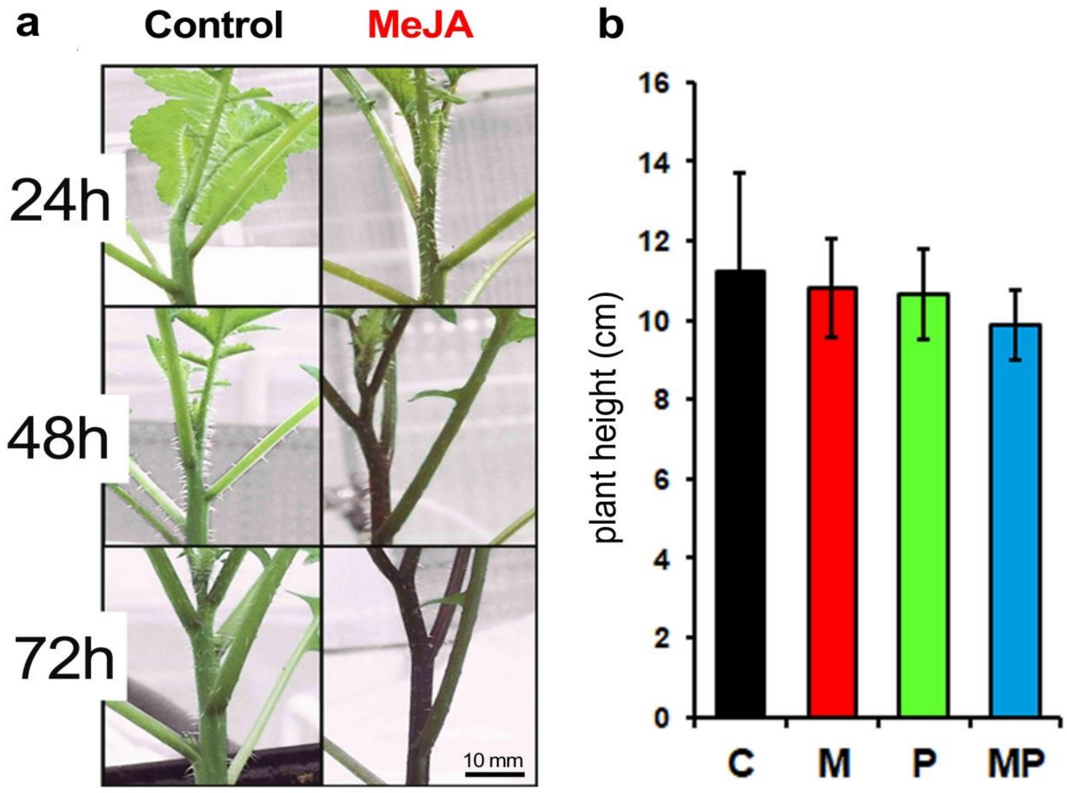

Figure S1. Induced plant phenotypes by MeJA and caterpillar herbivory. **a.** Effects of MeJA (1mM) treatment on stem *B. nigra,* showing induced pigmentation at 24, 48, and 72 hours after MeJA application. **b.** Average plant height measured after exposure to single and sequential treatments, abbreviations: controls (C), MeJA (M), *P. brassicae* herbivory (P), and sequential MeJA and herbivory (MP). Error bars = S.E.

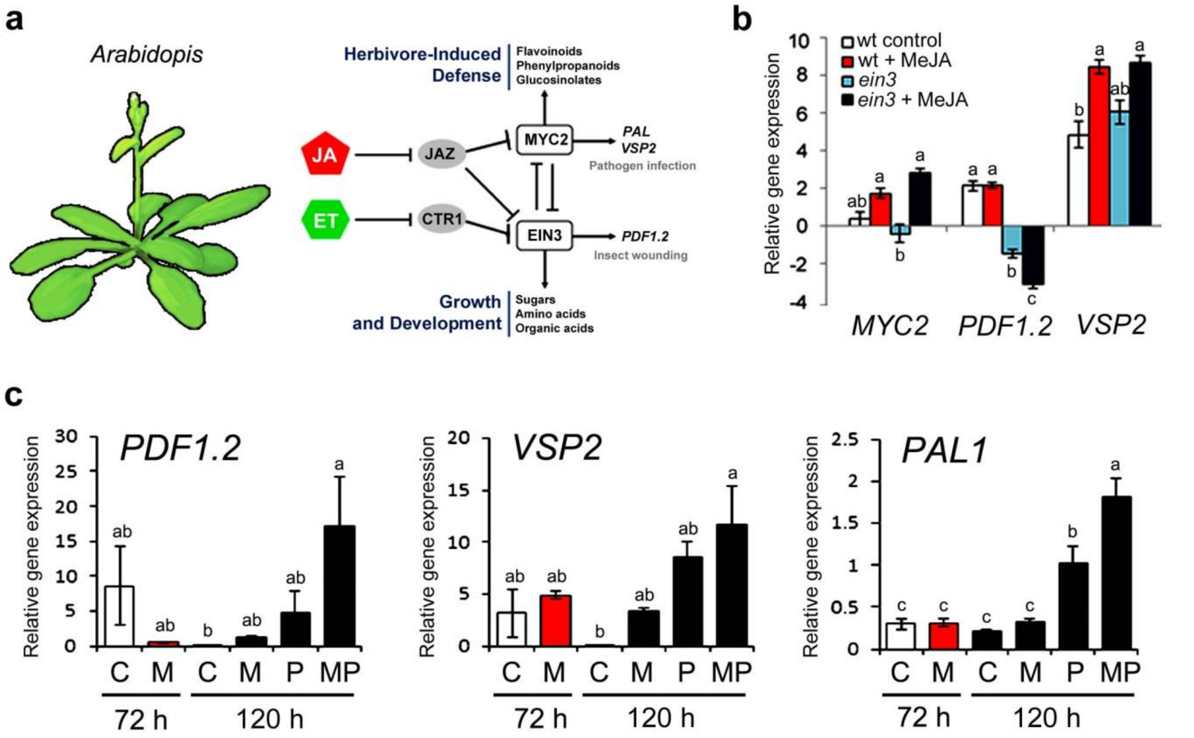

Figure S2. Comparative gene expression analysis in *Arabidopsis.* **a.** MeJA regulation of herbivore-induced responses in the model plant *A. thaliana*, showing fine-tuning of defence responses along the JA/ET pathways via antagonistic interaction between *MYC2* and *EIN3.* **b.** Cross-talk interactions between the JA and ET pathways were induced by treatment with MeJA (1mM, 72 h), resulting in the up-regulation of *MYC2* (JA pathway) in *ein3-1* mutants, and up-regulation of *VSP2* with concurrent down-regulation of *PDF1.2* in *ein3-1* mutants, but not in wt (col-0) plants. **c.** When wt (col-0) plants were tested for the same responses after MeJA (1mM, 72 h), or following *P. brassicae* caterpillar herbivory (120 h), changes in the relative expression of *PDF1.2*, *VSP2* and *PAL1* (phenylalanine metabolism) showed no clear effects post initial MeJA treatment, while all genes were up-regulated after sequential caterpillar feeding Significance of differences between samples and treatments was tested with ANOVA (n=3; Tukey comparisons). Treatment abbreviations: controls (C), MeJA (M), herbivory (P), and sequential MeJA and herbivory (MP). Error bars = S.E.

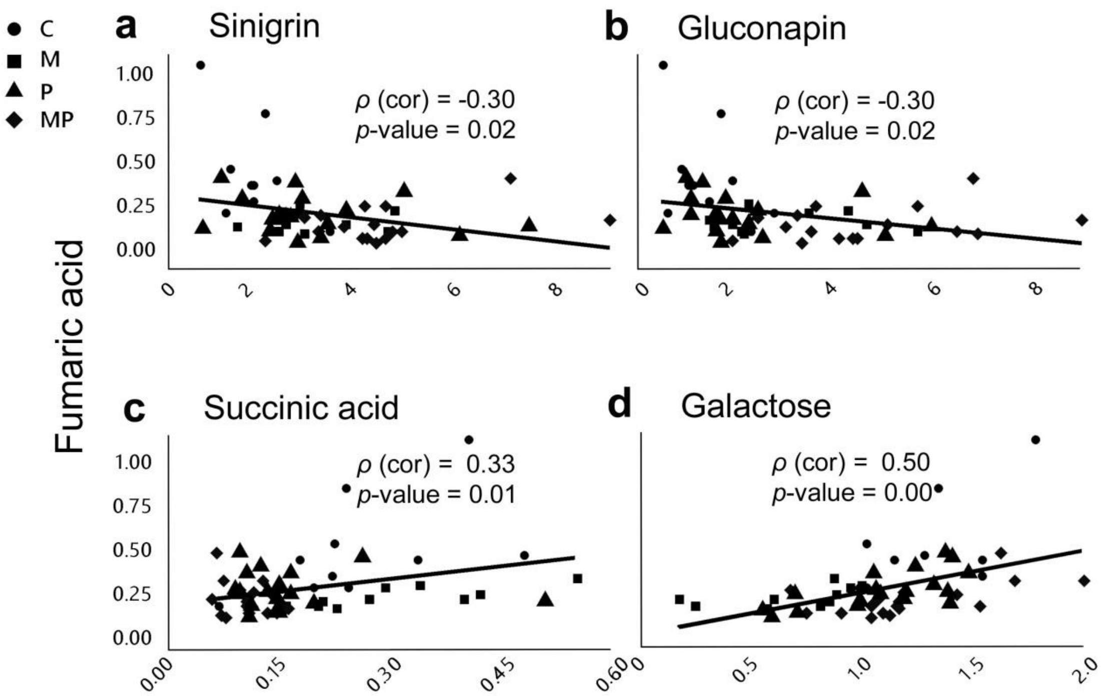

Figure S3. Metabolic correlation analysis. The TCA cycle intermediate metabolite, fumaric acid, is negatively correlated with specialized defence metabolites, e.g. GSLs (**a,b**) and positively correlated other primary central metabolites (**c,d**). Comparative correlation analysis further indicated response-specific internal metabolic reconfiguration across treatments (see Table S4). Pearson´s correlation coefficient and *P*-value. Treatment abbreviations: controls (C), MeJA (M), herbivory (P), and sequential MeJA and herbivory (MP).

Table S1. Growth and defence metabolism differences across leaf ages.

Metabolic changes as measured by GC-TOF-MS in leaves of *B. nigra*, 72 hours after MeJA application, assessed according to leaf position (top (L1-L3), mid (L4-L6), and bottom (L7-L8).. Values are (%) change relative to controls. Blue and red colors indicate up- and down- regulation. Significant *P*-values (< 0.05, two-tails student t-test) are highlighted in yellow (Fig.1 in main article).

	% change			*P*-value		
	**Bottom**	**Mid**	**Top**	**Bottom**	**Mid**	**Top**
**Amino acids**						
Leucine	52.0	12.0	105.0	0.070	0.449	0.040
Phenylalanine	68.3	21.5	9.8	0.015	0.249	0.732
Aspartic acid	5.7	-17.5	-25.5	0.826	0.309	0.029
**TCAs**						
Phosphate	46.9	46.0	23.0	0.013	0.006	0.066
Citric acid	68.5	115.6	66.7	0.016	0.000	0.018
α-ketoglutaric acid	350.8	223.5	121.7	0.016	0.017	0.019
Succinic acid	139.1	95.5	10.5	0.000	0.001	0.635
Fumaric acid	-32.9	-30.8	-89.5	0.036	0.009	0.038
Oxaloacetic acid	Nd	151.6	151.0	0.350	0.206	0.010
**Organic acids**						
Malic acid, 2-methyl	70.1	61.9	36.9	0.011	0.004	0.287
3-oxoglutaric acid	237.5	153.4	85.0	0.008	0.005	0.011
Galacturonic acid	125.1	124.1	180.5	0.058	0.001	0.042
Gluconic acid	68.3	67.1	38.7	0.010	0.006	0.021
Glucoronic acid	85.2	28.7	-10.7	0.027	0.148	0.545
**Antioxidants**						
Ascorbic acid	82.4	47.1	13.7	0.001	0.014	0.428
Dehydroascorbic acid	156.0	89.3	165.8	0.000	0.000	0.000
**Lipids**						
α-Linolenic acid	0.7	-12.9	-36.9	0.946	0.206	0.002
Heptadecanoic acid	49.4	24.0	12.1	0.046	0.011	0.249
Lauric acid	41.6	13.1	38.2	0.083	0.065	0.000
**Sugars**						
Fructose	-53.5	-57.2	-82.9	0.062	0.024	0.002
Sorbose	-48.6	-50.2	-76.6	0.073	0.031	0.003
Fructose-6-P	60.5	18.8	-15.2	0.003	0.224	0.244
Glucose	78.6	28.2	-6.4	0.025	0.112	0.683
Glucose 6-P	57.8	16.5	-8.2	0.003	0.245	0.511
Mannose	78.5	28.5	-6.6	0.025	0.109	0.674
Rhamnose	78.3	41.0	-8.1	0.011	0.078	0.696
Galactose	65.2	21.5	-6.3	0.017	0.121	0.616
Trehalose	164.0	158.5	112.1	0.000	0.000	0.001
Maltose	162.0	160.1	113.4	0.000	0.001	0.001
Xylose	35.4	47.0	45.5	0.044	0.000	0.001
Xylulose-5-P	81.9	64.0	77.8	0.004	0.008	0.000
Xylobiose	134.6	2.3	74.2	0.018	0.883	0.018
Erythrose	39.4	17.3	14.5	0.152	0.142	0.334
myo-Inositol	60.3	90.9	177.8	0.052	0.014	0.000
Mannitol	84.1	28.6	-7.3	0.025	0.117	0.651
Xylitol	18.6	35.2	44.1	0.349	0.039	0.005
**Phenolics**						
Caffeic acid	-5.7	-24.7	-40.3	0.574	0.043	0.019
Shikimic acid	148.0	140.9	54.8	0.015	0.016	0.108
Salicylic acid	11.4	60.3	-32.6	0.216	0.337	0.022
α-Tocopherol	3.9	25.6	83.5	0.851	0.211	0.000
**Amines**						
Ethanolamine	-1.0	-10.0	-35.7	0.971	0.606	0.039
Mannosamine, acetyl	40.6	37.1	79.6	0.019	0.027	0.001
Glucosamine, acetyl	45.4	32.9	71.2	0.017	0.051	0.003

Table S2. Plant metabolic responses to MeJA and herbivory.

Class	Metabolites	VIP[LV1]	VIP[LV2]	(P)	(M)
Amino acids	Aspartic acid	1.99	-	↓	
	Phenylalanine	1.99	-	↓	
	Tryptophan	1.46	1.19	↓	↓
	Alanine	1.28	-	↓	
	AABA Butyric acid, 2-amino-	1.09	-	↓	
	Threonine	1.07	1.20	↓	
TCAs	Pyruvic acid	1.03		↑	
	Citric acid	1.24	1.00	↑	↑
	Aconitic acid, *cis-*	1.87	1.42	↑	↑
	α-Ketoglutaric acid	1.62	1.23	↑	↑
	Succinic acid	1.57	1.45	↓	↑
	Fumaric acid	1.49	1.20	↓	↓
	Malic acid	1.55	1.26	↓	↓
Organic acids	Galactonic acid	1.83	1.46	↑	↑
	Maleic acid	1.64	1.30	↓	↓
	Oxalic acid	1.37	1.36	↓	↓
Antioxidants	Dehydroascorbic acid	1.64	1.19	↑	↑
	Ascorbic acid	1.54	1.22	↑	↓
	Glutathione oxidized (GSSG)	1.53	1.66	↓	↓
Lipids	α-Linolenic acid	1.43	1.09	↓	↓
	Myristic acid	2.26	1.77	↑	↑
Sugars	Sucrose	2.06	-	↑	
	Fructose	1.86	1.63	↓	↓
	Glucose	-	1.83		↓
	Gentiobiose	1.39	1.50	↑	↓
	Ribose	-	1.71		↓
	Inositol, *myo-*	1.56	1.07	↑	↑
	Sorbitol	-	2.08		↑
	Trehalose	1.22	1.33	↑	↑
	Galactose	-	1.06		↓
Glucosinolates	Gluconapin	1.62	1.37	↑	↑
	Sinigrin	1.53	1.44	↑	↑
	Glucoerucin	1.26	-	↑	
	Glucotropaeolin	1.07	1.34	↑	↑
	6-methylthio-3-oxohexyl-GSL	1.01	-	↑	
Hydroxycinnam der.	Disinapoylgentiobiose	1.22	-	↑	
	1-Caffeoyl-beta-D-glucose	1.13	1.10	↓	↓
	1-*O*-sinapoylglucose	1.12	1.57	↓	↓
	4-*O*-beta-D-glucosyl-sinapate	1.00	-	↑	
	1-2-disinapoylglucoside	1.00	-	↑	
	Sinapic acid	-	1.27		↓
	p-Coumaroyl-D-glucose	-	1.14		↑
	Feruloylmalic acid	-	1.13		↑
Flavonoids	Qn-3-sinapoylsophoroside-7-glucos.	1.02	1.11	↑	↓
Unknowns	148	1.13	1.06	↑	↓
	381	1.03		↑	

Metabolite contribution for components LV1 and LV2 (PLS-DA, four components; $R^2X(cum)$ = 42%, $R^2Y(cum)$ = 75%, $Q^2(cum)$ = 56%; see Fig.3a) of *B.nigra* responses towards herbivory by *P. brassicae* caterpillars and MeJA treatment, respectively. The significant contribution to the model for each metabolite is reported as their respective variable importance in the projection (VIP) scores >1.00. Metabolites are ordered from higher to lower VIP values following the component LV1, and their coefficients are reported as increased (blue ↑) or decreased (red ↓) abundance relative to treatment.

Table S3. Enhanced plant metabolic responses to sequential MeJA treatment and caterpillar herbivory.

**Metabolites**	**Class**	**VIP** (MP vs. P)	
Glucotropaeolin	Glucosinolate	1.93	↑
Gluconapin	Glucosinolate	1.80	↑
Maltotriose	Sugar	1.78	↑
Sucrose	Sugar	1.74	↑
Feruloylmalic acid	Hydroxycinnamate	1.66	↑
cis-Aconitic acid	TCA	1.63	↑
Flavanone (putative)	Flavonol glucoside	1.60	↑
Sinigrin	Glucosinolate	1.52	↑
Galactonic acid	Organic acid	1.51	↑
381	Unknown	1.51	↑
Neoglucobrassicin	Glucosinolate	1.48	↑
Caffeoyl-glucoside (putative)(1)	Hydroxycinnamate	1.39	↑
p-coumaroyl-D-glucose	Hydroxycinnamate	1.37	↑
Glucoerucin	Glucosinolate	1.32	↑
3-methylbutyl-GSL	Glucosinolate	1.22	↑
Caffeoyl-glucoside (putative (2)	Hydroxycinnamate	1.17	↑
Disinapoylgentiobiose	Hydroxycinnamate	1.15	↑
Inositol, *myo-*	Sugar	1.14	↑
Dehydroascorbic acid	Redox	1.09	↑
Sinalbin	Glucosinolate	1.02	↓
α-Linoleic acid	Lipid	1.03	↓
Qn-3-sinapoylsophoroside-7-glucoside	Flavonol glucoside	1.04	↓
Km 3-sinapoylsophoroside-7-glucoside	Flavonol glucoside	1.04	↓
4-hydroxyglucobrassicin	Glucosinolate	1.04	↓
Succinic acid	TCA	1.06	↓
Glucoraphanin	Glucosinolate	1.06	↓
Km-3-hydroxyferuloylsophoroside-7-	Flavonol glucoside	1.07	↓
Qn-glucoside (putative)	Flavonol glucoside	1.07	↓
Sinapoylhydroxyferuloylgentiobiose	Hydroxycinnamate	1.07	↓
Qn-3-sophorotrioside-7-glucoside	Flavonol glucoside	1.11	↓
Trisingentiobiose	Hydroxycinnamate	1.12	↓
Qn-7-sophoroside	Flavonol glucoside	1.14	↓
Km 3-sinapoylsophoroside (putative)	Flavonol glucoside	1.16	↓
Km-3-*p*-coumaroylsophoroside-7-glucos.	Flavonol glucoside	1.16	↓
Qn-3-sinapoylsophoroside-7-glucos.	Flavonol glucoside	1.17	↓
Km-3-hydroxyferuloyldiglucoside-	Flavonol glucoside	1.17	↓
Malic acid	TCA	1.21	↓
421	Unknown	1.22	↓
Threonic acid	Redox	1.28	↓
Km-glucoside (putative)	Flavonol glucoside	1.32	↓
Tryptophan	Amino acid	1.34	↓
1-O-sinapoylglucose	Hydroxycinnamate	1.47	↓
Fructose	Sugar	1.53	↓
1-caffeoyl-β-D-glucose	Hydroxycinnamate	1.64	↓
Glutathione (GSSG)	Redox	1.66	↓
Oxalic acid	Organic acid	1.67	↓

Metabolite contribution the effect of MeJA treatment on *B.nigra* responses towards sequential herbivory by *P. brassicae* (OPLS-DA model; 1+1+0; R^2Xcum = 28%, R^2Y cum = 86%, Q^2 cum = 70%). The significant contribution to the model for each metabolite is reported as their respective variable importance in the projection (VIP) scores >1.00. Metabolites are ordered from higher to lower VIP values, for increased (blue ↑) and decreased (red ↓) abundance in plants pre-treated with MeJA (MP) compared to plants only treated with herbivory (P).

Table S4. Central metabolic correlations in response to MeJA and herbivory.

Comparative correlation (Steuer, 2006) between the central TCA intermediate metabolite fumaric acid, with central metabolites and specialized metabolites, including PPs and GSLs, evaluated across all MeJA and herbivory treatments.

	corr.	**All treat.**		**C**		**M**		**P**		**MP**	
**Fumaric acid**	Succinic acid	0.33	*0.01*	0.52	*0.12*	-0.32	*0.17*	-0.06	*0.81*	-0.37	*0.21*
	Galactose	0.50	*0.00*	0.62	*0.05*	0.61	*0.00*	0.67	*0.00*	0.61	*0.00*
	Xylose	0.40	*0.00*	0.65	*0.04*	0.17	*0.64*	0.26	*0.29*	0.65	*0.00*
	Ribose	0.04	*0.74*	0.67	*0.03*	0.12	*0.73*	0.26	*0.28*	0.52	*0.03*
	Glycerol	0.25	*0.06*	0.66	*0.04*	0.18	*0.62*	-0.15	*0.55*	0.30	*0.25*
	Glycerol-3-P	-0.36	*0.01*	-0.67	*0.06*	0.01	*0.96*	-0.10	*0.68*	-0.45	*0.07*
	Sinigrin	-0.30	*0.02*	-0.61	*0.06*	0.41	*0.11*	-0.22	*0.39*	0.41	*0.11*
	Gluconapin	-0.30	*0.02*	-0.45	*0.19*	0.28	*0.27*	-0.20	*0.42*	0.28	*0.27*
	Glucoerucin	-0.21	*0.10*	-0.56	*0.09*	0.22	*0.38*	-0.08	*0.73*	0.22	*0.38*
	4-hydroxygluc	-0.08	*0.52*	-0.15	*0.64*	0.28	*0.44*	-0.37	*0.12*	0.55	*0.02*
	p-coumaroyl-gl	-0.22	*0.10*	-0.71	*0.02*	0.62	*0.06*	-0.45	*0.06*	-0.19	*0.47*
	Qn-3-sin-7gluc	-0.10	*0.49*	-0.60	*0.07*	0.42	*0.23*	0.20	*0.42*	0.31	*0.23*

Fumaric acid correlation with primary central metabolites (yellow) and specialized defence metabolites (green). Treatment abbreviations: controls (C), MeJA (M), herbivory (P), and sequential MeJA and herbivory (MP). Values reported in matrix as: Pearson correlation and *P-value*. Blue and red colors indicate positive and negative correlation. Significant *P*-values (< 0.05) are highlighted in yellow. See also Fig.S3.

Supplementary Methods

Gene expression analysis in *Arabidopsis*

A supplementary experiment was set up with the ethylene insensitive (*ein3-1*) *Arabidopsis* mutant to study jasmonic acid (JA) and ethylene (ET) hormonal cross talks associated with senescence and bio-stress defences ((Chao *et al*., 1997). MeJA was applied as described for *B. nigra*, and rosettes sampled to study gene expression of selected genes: *MYC2* (transcription factor, Song *et al*., 2014), *VSP2* (*VEGETATIVE STORAGE PROTEIN 2*) and *PDF1.2* (*PLANT DEFENSIN 1.2*) (Manners *et al*., 1998; Liu *et al*., 2005; Verhage *et al*., 2011) (C and M; N=3 per treatment). An additional time-series experiment was conducted with use of only wild types (Col-0; C and M, 18 replicates each). Plants were harvested after 72 h (time point 1), C and M, 6 plants per treatment) and the remaining plants exposed to herbivory by six first instar *P. brassicae* caterpillars (P and MP). After a 120 h (time point 2), the remaining 24 plants were sampled (C, M, P and MP; six replicates each) as described before and used to characterize the expression of *PDF1.2*, *VSP2*, and *PAL1* (*PHENYLALANINE AMMONIA LYASE 1*) (Yan *et al*., 2013) (**Fig.S2**).

RT-qPCR:

RNA was extracted according to Chang *et al*. (1993) and DNase treated (DNA-free™ Kit; Ambion) followed by quantification with a ND-1000 NanoDrop spectrophotometer (NanoDrop Technologies, Wilmington, DE, USA). cDNA synthesis was performed on 0.5 µg RNA using iScript™ cDNA synthesis kit (Bio-Rad). RT-qPCR analyses were performed with primers for *MYC2*, *VSP2*, *PDF1.2,* and *PAL1*. LightCycler® 480 SYBR Green I Master (Roche Diagnostics GmbH, Mannheim, Germany) mix was used with five times dilutions of cDNA template with 0.5 pmol of each primer using a Bio-Rad CFX96 Real Time System. The PCR cycles were: initial denaturation at 95°C 3 min; denaturation at 95°C for 10 s, primer annealing at 58°C for 10 s, and extension at 72°C for 20 s for 40 cycles. The average *Cq* value of the *GAPDH* and *SAND* reference genes was subtracted from the corresponding *Cq* value for each gene to obtain a normalized *ΔCq* value. The relative expression levels compared to controls were calculated from the formula $2^{-\Delta Ct}$ and data were log transformed.

<u>*RT-qPCR primers:*</u>

MYC2 (AT1g32640)
Forward: AACCACGTCGAAGCAGAGAGAC
Reverse: TTGGTACAACCGCTCGTACGC

VSP2 (AT5g24770)
Forward: TTGGCAATATCGGAGATCAAT
Reverse: GGGACAATGCGATGAAGATAG

PDF1.2 (AT5g44420)
Forward: CTTGTTCTCTTTGCTGCTTTCGAC
Reverse: TTGGCTCCTTCAAGGTTAATGCAC

PAL1 (AT2g37040)
Forward: GCAGTGCTACCGAAAGAAGTGG
Reverse: ATCCTGTTCGGGATAGCCGATG

GAPDH (AT1g13440)
Forward: TTGGTGACAACAGGTCAAGCA
Reverse: AAACTTGTCGCTCAATGCAATC

SAND (AT2g28390)
Forward:GCCTGAACCGTCTTCTGTGGAGT
Reverse: CTCAATCTCAGACACACTGGTGCTA

References for Supplementary Methods

Chang, S., Puryear, J., Cairney, J. (1993). A simple and efficient method for isolating RNA from pine trees. *Plant Molecular Biology Reporter*, *11*(2), 113-116.

Chao, Q., Rothenberg, M., Solano, R., Roman, G., Terzaghi, W., Jr., E. (1997). Activation of the ethylene gas response pathway in *Arabidopsis* by the nuclear protein *ETHYLENE-INSENSITIVE3* and related proteins. *Cell*, *89*(7), 1133-1144.

Liu, Y., *et al.* (2005). *Arabidopsis* vegetative storage protein is an anti-insect acid phosphatase. *Plant physiology*, *139*(3), 1545-1556.

Manners, J. M., *et al.* (1998). The promoter of the plant defensin gene *PDF1.2* from *Arabidopsis* is systemically activated by fungal pathogens and responds to methyl jasmonate but not to salicylic acid. *Plant Molecular Biology*, *38*(6), 1071-1080.

Sar, M., Chauvin, A., Pascaud, F., Kellenberger, S., Ee., F. (2013). *GLUTAMATE RECEPTOR-LIKE* genes mediate leaf-to-leaf wound signalling. *Nature*, *500*(7463), 422-426.

Song, S., Huang, H., Gao, H., *et al.* (2014). Interaction between *MYC2* and *ETHYLENE INSENSITIVE3* modulates antagonism between jasmonate and ethylene signaling in *Arabidopsis*. *The Plant Cell*, *26*(1), 263-279.

Verhage, A., *et al.* (2011). Rewiring of the jasmonate signaling pathway in *Arabidopsis* during insect herbivory. *Frontiers of Plant Science*, *2*, 47.

Yan, L., *et al.* (2013). The heterologous expression in *Arabidopsis thaliana* of sorghum transcription factor *SbbHLH1* downregulates lignin synthesis. *Journal of Experimental Botany*, *64*(10), 3021-3032.
